# The Intrathymic Pathogenesis of Myasthenia Gravis

**DOI:** 10.1080/17402520400001769

**Published:** 2004

**Authors:** Arnold I. Levinson, Decheng Song, Glen Gaulton, Yi Zheng

**Affiliations:** 1 Allergy and Immunology Section University of Pennsylvania School of Medicine RoomBRB II/III Philadelphia PA USA; 2 Department of Pathology and Laboratory Medicine University of Pennsylvania School of Medicine RoomBRB II/III Philadelphia PA USA

## Abstract

The thymus is considered to play an important role in the pathogenesis of Myasthenia gravis, an autoimmune disease characterized by antibody-mediated skeletal muscle weakness. However, its role is yet to be defined. The studies described herein summarize our efforts to determine how intrathymic expression of the neuromuscular type of acetylcholine (ACh) receptors is involved in the immunopathogenesis of this autoimmune disease. We review the work characterizing the expression of neuromuscular ACh receptors in the thymus and advance a new hypothesis that examines the intrathymic expression of this autoantigen in disease pathogenesis.


*Clinical & Developmental Immunology*, September/December 2004 Vol. 11 (3/4), pp. 215–220

Taylor & Francis healthsciences

### The Intrathymic Pathogenesis of Myasthenia Gravis

ARNOLD I. LEVINSON[a,*], DECHENG SONG[a], GLEN GAULTON[b] and YI ZHENG[a]

[a]*Allergy and Immunology Section, University of Pennsylvania School of Medicine, Room 1014 BRB II/III, 421 Curie Boulevard, Philadelphia, PA 19104, USA;* [b]*Department of Pathology and Laboratory Medicine, University of Pennsylvania School of Medicine, Room 357 BRB II/III, 421 Curie Boulevard, Philadelphia, PA 19104, USA*

The thymus is considered to play an important role in the pathogenesis of Myasthenia gravis, an autoimmune disease characterized by antibody-mediated skeletal muscle weakness. However, its role is yet to be defined. The studies described herein summarize our efforts to determine how intrathymic expression of the neuromuscular type of acetylcholine (ACh) receptors is involved in the immunopathogenesis of this autoimmune disease. We review the work characterizing the expression of neuromuscular ACh receptors in the thymus and advance a new hypothesis that examines the intrathymic expression of this autoantigen in disease pathogenesis.

*Keywords*: Myasthenia gravis; Thymus; Acetylcholine receptor; Intrathymic expression

### INTRODUCTION

Myasthenia gravis (MG) is a disease characterized by weakness of striated muscles. The weakness is due to impaired neuromuscular transmission resulting from a reduction of the number of receptors for the neurotransmitter, acetylcholine (ACh) at the postsynaptic myoneural junction. This reduction is caused by the action of anti-acetylcholine receptor (anti-AChR) antibodies, reviewed in Levinson *et al*. (1987). MG is a prototypic autoimmune disease; the immune effector mechanisms and autoantigenic target have been delineated. However, the events leading to the abrogation of self-tolerance to the neuromuscular type of AChR (nAChR) remain a mystery. The thymus gland has long been considered to hold the key to solving this mystery, although the nature of its involvement remains to be elucidated.

Interest in a pathogenic role for the thymus in MG has been fueled by pathologic, clinical and immunologic lines of evidence as reviewed by Levinson and Wheatley (1995). Briefly, thymus glands of 60–70% of MG patients demonstrate the histological pattern of germinal center hyperplasia whereas another 10% display cortical epithelial cell thymomas. Thymectomy, particularly in young patients (<40 years of age) with thymic hyperplasia, is followed by clinical improvement and remains a first-line therapeutic intervention. nAChR-specific B and T cells have been recovered from MG thymus specimens but not control thymus tissue. This indicates that the autoimmune effector cells populate the diseased thymus.

### EXPRESSION OF nAChR IN THYMUS

A major feature of the thymus that very likely represents an important pathogenic link to MG is the expression of nAChRs on cells in this organ as reviewed in Levinson and Wheatley (1995). The issue of thymic expression of AChR has attracted considerable interest, in part, because of the pivotal role that self-antigen expression in the thymus plays in tailoring the T cell repertoire. Moreover, the expression of nAChRS on thymic cells represents the first description of the “promiscuous” intrathymic expression of an organ-specific self-antigen that is the target of an autoimmune attack in the periphery. The early reports prompted some investigators to propose that the thymus might actually serve as a site of sensitization for this autoantigen (Wekerle *et al.*, 1978). Taken at face value, this idea might be viewed as being incongruent with the cardinal immunologic construct, noted above, namely that thymic self-proteins, particularly those expressed on epithelial cells orchestrate the induction of self-tolerance (Klein and Kyewski, 2000).

The nAChRs are expressed in two major forms as reviewed in Levinson (2001a). The so-called mature or junctional form is expressed on innervated muscles and the immature or fetal form is expressed on non-innervated tissue. At the mature (innervated) myoneural junction, nAChRs are comprised of four subunits labeled α, β, δ and ε. Two alpha subunits and one each of the other subunits are assembled, like the whalebone in a corset, to form an asymmetric hourglass channel spanning the membrane. Two alternatively spliced alpha subunit

*Corresponding author. E-mail: cwiuitt@mail.med.upenn.edu

ISSN 1740-2522 print/ISSN 1740-2530 online © 2004 Taylor & Francis Ltd
DOI: 10.1080/17402520400001769

isoforms have been characterized, P3A$^-$ and P3A$^+$. The larger P3A$^+$ isoform, which includes an additional sequence of 25 amino acids between exons 3 and 4, is found only in humans and other primates. In fetal muscle, as in adult denervated muscle or nonjunctional membrane, a γ subunit replaces the ε subunit found in mature, innervated muscle endplates.

In the analysis of nAChRs in the thymus, many investigators have focused on the nAChR alpha subunit (nAChRα) since it is the source of the important pathogenic T and B cell epitopes for the pathogenic autoimmune response in MG (Oshima *et al.*, 1990; Zhang *et al.*, 1990; Conti-Fine *et al.*, 1998; Fuji and Lindstrom, 1988). Expression of this subunit was originally reported on a variety of thymic cells including epithelial cells (Engel *et al.*, 1977), thymocytes (Fuchs *et al.*, 1980), and myoid cells (Kao and Drachman, 1977; Schluep *et al.*, 1987). Myoid cells, which share phenotypic properties with skeletal muscle cells, were originally viewed as the principal AChR-expressing cells in thymus (Schluep *et al.*, 1987). They are found in the medulla of both normal and MG thymus.

In the past several years, there has been renewed interest in thymic cells as a source of nAChR expression. Several investigators, including ourselves, have taken a molecular approach in characterizing thymic AChRs and identifying cell populations expressing them. Using reverse-transcription-PCR (RT-PCR) technology, we reported that mRNA for the AChRα was expressed in normal mouse (Wheatley *et al.*, 1992), normal human and MG thymus (Wheatley *et al.*, 1993; Zheng *et al.*, 1999). We also reported that AChRα mRNA was expressed on transformed murine thymic cortical and medullary epithelial cell lines and thymic dendritic cell lines (Wheatley *et al.*, 1992). We found that mRNAs encoding both major isoforms of the human AChRα, i.e. P3A$^+$ and P3A$^-$, were expressed in normal and MG thymus and normal human thymic epithelial cells (Wheatley *et al.*, 1993; Zheng *et al.*, 1999). Sequencing of P3A$^+$ and P3A$^-$ cDNA clones recovered from control and MG thymus indicated that they share the same nucleotide sequence as their respective counterparts at the myoneural junction. Thus, unless there are posttranslational changes, the structure of the AChRα proteins expressed in the thymus and the periphery are likely to be identical. These results provided a structural basis for proposing that an immune response directed at thymic nAChRα may be responsible for initiating or perpetuating disease. Berrih-Aknin subsequently reported that AChRα protein as well as mRNA was expressed on human thymic epithelial cells (Wakkach *et al.*, 1978).

However, there is still a controversy over the expression of the other subunits on thymic cells and whether they are expressed as components of intact receptors. Some of the reported discrepancies may reflect differences in the ages of the thymus donors and differences in the design of the RT-PCRs. At this time, it appears as if ε and β mRNAs are expressed in most normal and MG thymus specimens with variable expression of δ and γ subunits (Naveneetham *et al.*, 2001; Bruno *et al.*, 2004). Expression of the AChR subunits appears to be concentrated in the thymic medullary compartment.

To gain a better understanding of how intrathymic expression of nAChRα might be linked to the development of disease, we addressed additional features of nAChRα expression in the thymus. We observed that the smaller P3A$^-$ isoform is present in approximately a five-fold excess in both MG and control thymic tissue and a 2.5-fold excess in a non-transformed human thymic epithelial cell (TEC) line relative to the larger P3A$^+$ isoform (Fig. 1) (Zheng *et al.*, 1999). The greater expression of the P3A$^-$ isoform in thymus does not parallel its expression in healthy and MG muscle tissue where both isoforms show equivalent expression (Beeson *et al.*, 1990). These observations suggest that the expression of mRNAs encoding the P3A$^-$ and P3A$^+$ isoforms is regulated differently in human thymus and muscle. Since the same disproportionate expression of P3A$^-$ was observed in control and MG thymus, it appears that the differential pattern of expression observed in thymus relative to muscle is not a manifestation of thymic pathology in MG. Rather, this pattern may reflect control processes that are unique to these two distinct tissue compartments. Presently, it is not known whether the disproportionate expression of P3A$^-$ vs. P3A$^+$ isoforms has pathogenic significance.

We also observed that expression of both P3A$^+$ and P3A$^-$ mRNAs are increased in MG thymus compared to control thymus (Fig. 2) (Zheng *et al.*, 1999). This finding parallels that reported for skeletal muscle where AChR mRNA expression was found to be greater in MG muscle than in control muscle (Guyon *et al.*, 1993). The finding of increased AChRα mRNA expression in MG thymus may represent an attempt to compensate for the destructive action of locally secreted anti-AChR antibodies. It is also possible that the increased AChR mRNA expression may reflect the antecedent action of other local environmental factors, e.g. cytokines.

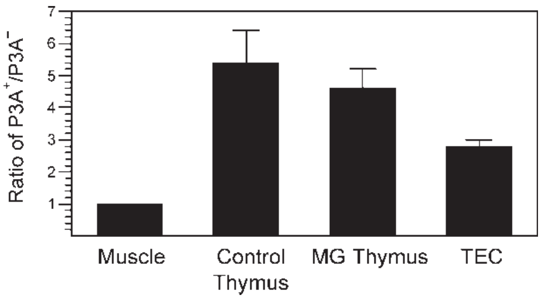

FIGURE 1 Relative expression of AChRα P3A$^-$ and P3A$^+$ isoforms in thymus and TEC. Compilation of data from 14 MG thymuses, 7 control thymuses, and 4 separate TEC experiments. The signals for the P3A$^-$ and P3A$^+$ bands on Southern blots were quantitated on a phosphorimager. The P3A$^-$/P3A$^+$ ratios are shown. Expression of P3A$^-$ exceeded that of P3A$^+$ by a factor of 5.5 ± 0.9 (mean ± SEM) in Control thymus, 4.7 ± 0.05 in MG thymus, and 2.8 ± 0.2 in TEC. (Copyright: *Clinical Immunology*, 1:1999).

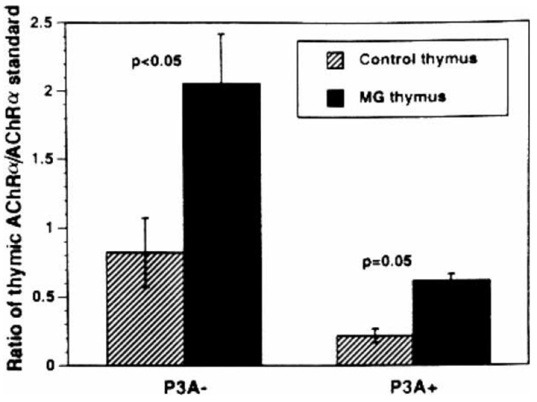

FIGURE 2 Semiquantitative RT-PCR: compilation of results from thymus specimens of seven control subjects and fourteen MG patients. The signal intensity of the AChRα bands is normalized to the signal intensity of the standard by calculating the ratio of thymic AChRα/AChRα standard. The expression of $P3A^-$ and $P3A^+$ isoforms in MG thymus is 2.5- and 2.8-fold greater, respectively, than that in control thymus. (Copyright: *Clinical Immunology*, 1:1999).

### REGULATION OF INTRATHYMIC nAChRα EXPRESSION

IL-1 and IL-6 production by epithelial cells is increased in hyperplastic thymic tissue obtained from MG patients compared to thymus from control subjects (Cohen-Kaminsky *et al.*, 1978; Emilie *et al.*, 1991). Since cytokines produced *in vitro* by thymic epithelial cell (TEC) lines demonstrate autocrine function (Galy and Spits, 1991), it seemed plausible that these cytokines or perhaps others produced by cells in the thymus might regulate TEC expression of AChR. We examined this possibility by incubating a human non-transformed TEC line with either IL-1β, IL-4, IL-6 and interferon-γ (IFN-γ). We found that neither IL-1, IL-4, nor IL-6 altered the expression of AChRα mRNA by this cell line (Zheng *et al.*, 1999). By contrast, IFN-γ increased expression of the $P3A^-$ and $P3A^+$ isoforms by factors of 2.7 and 2.8, respectively. It is known that IFN-γ up-regulates the expression of MHC class II antigens on thymic epithelial cells (Berrih-Aknin *et al.*, 1985; Galy and Spits, 1991). This dual effect of IFN-γ on AChRα and MHC antigens raises the possibility that this cytokine, and perhaps others, may alter expression of thymic AChRα *in vivo* in a manner that leads to the development or perpetuation of MG. Before addressing this idea, it would be helpful to briefly review the role of self-antigen expression in thymus plays in the development of T cell tolerance and consider how the thymus could serve as a site of immune activation.

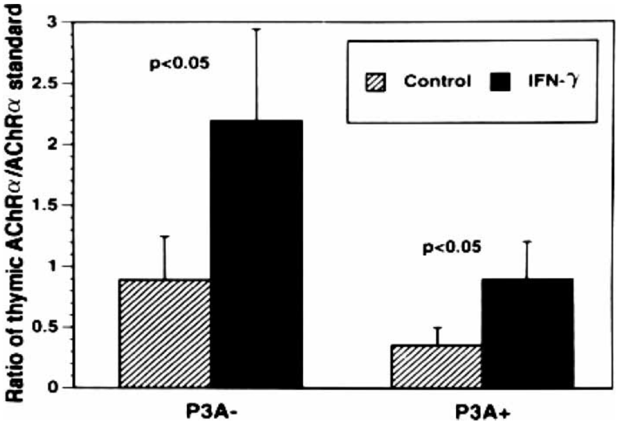

FIGURE 3 Semiquantitative RT-PCR: compilation of results from six TEC experiments depicting the effect of IFN-γ on expression of AChRα isoforms in TEC. To determine the effect of IFN-γ on the expression of AChRα $P3A^-$ and $P3A^+$ isoforms, we compared the normalized signal intensities of the isoforms (ratio of thymic AChRα/AChRα standard) detected in untreated and IFN-γ treated TEC9 cultures. Expression of $P3A^-$ mRNA was significantly greater in IFN-γ treated ($2.19 \pm 0.75$, mean + SEM) than in untreated cultures ($0.89 \pm 0.36$, $p < 0.05$, student's *t*-test). Likewise, expression of $P3A^+$ mRNA was significantly greater in IFN-γ treated ($0.9 \pm 0.31$) than in untreated cultures ($0.36 \pm 0.15$, $p < 0.05$). (Copyright: *Clinical Immunology*, 1:1999).

### INTRATHYMIC EXPRESSION OF SELF ANTIGEN AND THE DEVELOPMENT OF T CELL TOLERANCE

The thymus plays a fundamental role in the generation of the peripheral T cell repertoire as reviewed in Klein and Kyewski (2000), Sprent *et al.* (1988), Kisielow and Boehmer (1990), Alam *et al.* (1996) and Anderson *et al.* (1996). It is generally believed that thymocytes with low affinity receptors for self are positively selected for export to the peripheral lymphoid tissues where they comprise the T cell repertoire that recognizes exogenous antigens (Kisielow and Boehmer, 1990; Anderson *et al.*, 1996). By contrast, T cell tolerance to self is effected largely by the process of central deletion/inactivation. Developing thymocytes with high affinity receptors for self-peptide are silenced by apoptosis or anergy. There is widespread agreement that presentation of self-peptides by cortical epithelial cells is necessary for positive selection (Kisielow and Boehmer, 1990; Alam *et al.*, 1996; Anderson *et al.*, 1996). Thymic medullary epithelial cells and to a lesser extent, bone marrow-derived macrophages and dendritic are considered to be the major APCs involved in negative selection (Blackman *et al.*, 1990; Bonomo and Matzinger, 1993; Hugo *et al.*, 1994; Hoffmann *et al.*, 1995; Klein and Kyewski, 2000). However, central deletion is not complete even though a broad array of self-peptides is "promiscuously" expressed on medullary thymic epithelial cells (Klein and Kyewski, 2000). Self-reactive T cells escape from the thymus in small numbers, perhaps due to the fact that limiting levels of self-antigen limit the efficiency of tolerance induction (Adelstein *et al.*, 1991; Iwabuchi *et al.*, 1992; Oehen *et al.*, 1994). However, such self-reactive T cells are silenced by their anergic or ignorant status, i.e. they never encounter self-antigen in the periphery in a manner that leads to immune activation, or they are suppressed by regulatory T cells (Shevach, 2000).

### THE THYMUS AND T CELL TRAFFICKING

Based on the classic studies of Gowans, traffic of lymphocytes is generally considered to be unidirectional, i.e. out of the thymus into the blood and peripheral lymphoid organs (Gowans and Knight, 1964). However, small numbers of peripheral immunocompetent T cells migrate to the thymus, entering via the medulla (Naparstek *et al.*, 1982,1993; Michie *et al.*, 1988; Hirokawa *et al.*, 1989; Agus *et al.*, 1991; Gossmann *et al.*, 1991; Westermann *et al.*, 1991). Most of the thymic immigrants are T cells activated in the peripheral immune system although even resting T cells may gain access to the thymus (Hirokawa *et al.*, 1989; Agus *et al.*, 1991; Gossmann *et al.*, 1991; Westermann *et al.*, 1991). It is not known if the rate or number of thymic immigrants is increased by an inflammatory reaction in the thymus. Furthermore, it is not known if self-reactive T cell immigrants are activated when they encounter their specific antigens in the thymus. Thymus T cell immigrants specific for the lymphocytic choriomeningitis virus (LCMV) clear infectious foci from the thymus (Gossmann *et al.*, 1991). This observation indicates that peripheral T cells can be activated when they engage specific foreign antigens in the thymus in an appropriate context. When self-reactive T cells encounter their antigens in other tissue compartments in the presence of requisite co-stimulatory signals, they can be activated to express their differentiation program, reviewed in Mondino *et al.* (1996). One mechanism that leads to a milieu that promotes the abrogation of tolerance peripherally is infection. Local infection can lead to the upregulation of MHC antigens and co-stimulatory molecules on cells that express low levels of self-antigens and thereby lead to activation of autoreactive T cells (Mondino *et al.*, 1996).

### A NEW MODEL OF THYMIC INFLAMMATION AND ITS IMPACT ON "RETROGRADE" T CELL MIGRATION

Delineation of the molecular events, particularly in the thymus, that trigger MG has been hampered by the lack of a model system. Thymic pathology is not a feature of experimental models of MG in rodents. Although such models have provided insight into the pathogenesis of MG, they have not served to elucidate the role played by the thymus (Christadoss *et al.*, 2000). To address this issue we have developed a model of inflammation targeted to the thymic medulla, the site of thymic entry by peripheral T cells (Levinson *et al.*, 2001b). We generated molecular variants of the well characterized thymotrophic Gross murine leukemia virus (G-MLV), GD17, that had previously been shown to exclusively infect medullary thymic epithelium following their intrathymic injection in naïve mice. The variants were constructed to allow for easy casetting of a broad array of genes of interest. The thymo-tropic MLV vectors were created by ligating a 425 bp fragment containing the U3 region of GD-17 into the LTR backbone of the well defined M-MLV vector LXSH.

The vectors used in our studies are presented in linear form in Fig. 4. The parental LXSH vector includes 5′ M-MSV LTR, the psi packaging site and 5′ gag region, the hygromycin resistance gene under control of the SV40 promoter, and the 3′ LTR of M-MLV. For our experimental protocol, we modified this vector by insertion of the Lac z gene (LBSHG). We utilized LBSHG and LXSHG as our experimental and control vectors, respectively. As was true for GD17, we found that these vectors also target expression of encoded genes to the thymic medullary epithelium.

Balb/c mice were immunized to β-galactosidase (β-gal) and then injected intrathymically (i.t.) with the β-gal encoding vector LBSHG or the control vector LXSHG. Hematoxylin and eosin stained sections of thymus obtained four days after i.t. injection of LBSHG, but not LXSHG, showed obliteration of the cortical/medullary architecture with marked cellular expansion of the medulla. To determine whether this local inflammatory reaction non-specifically augmented the entry of peripheral T cells into the thymus, β-gal immunized mice were injected i.v. with a population of CFSE-labeled CD4+ T cells specific for an unrelated antigen four days after i.t. injection of LBSHG or LXSHG. The CD4+ T cells were derived from a transgenic mouse

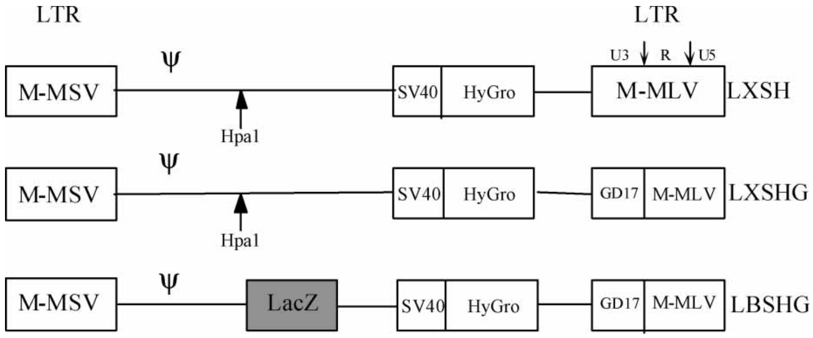

FIGURE 4 Schematic diagram of MLV-based vectors. The vectors used in these studies are presented in linear form. The parental LXSH vector includes the 5′ M-MCV LTR, the psi packaging site and the 5′ gag region, the hygromycin resistance gene under the control of the SV40 promoter, and the 3′ LTR of M-MLV. Vectors are modified by insertion of either GD-17 U3 or LacZ. (Copyright: *Annals of the New York Academy of Sciences*, 998:2003).

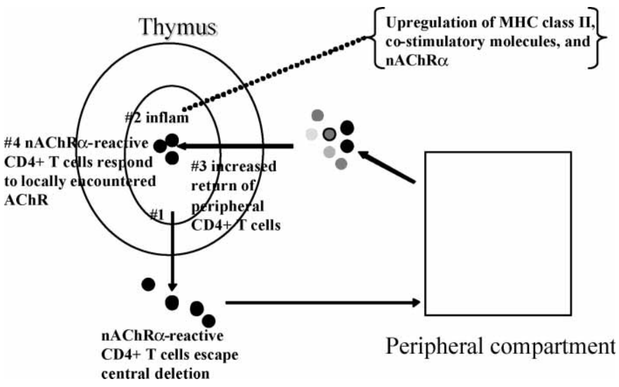

FIGURE 5 A new hypothesis bearing on the intrathymic pathogenesis of MG. Please see text for details. (Copyright: *Annals of the New York Academy of Sciences*, 998:2003)

bearing a T cell receptor that recognized an influenza hemagglutinin peptide. Animals that received LBSHG had 4.2-fold more CFSE-labeled CD4+ thymic immigrants than animals that received the control vector.

Using this model, we have begun to examine a new hypothesis bearing on the intrathymic pathogenesis of MG. (Fig. 5). The hypothesis posits that an inflammatory reaction to an unrelated antigen within the medulla of the thymus facilitates entry of peripheral AChRα-reactive CD4+ T cells that escaped central deletion. These cells enter the thymus in the medullary compartment where they encounter AChRα expressed on antigen presenting cells. The concomitant intrathymic inflammatory reaction creates a milieu that favors activation of these cells, i.e. upregulation of MHC class II antigens, co-stimulatory molecules on APCs, and perhaps upregulation of AChR expression. Presentation of AChRα epitopes to the CD4+ thymic immigrants leads to their activation, help for locally stimulated αAChR-reactive B cells, the production of anti-AChR antibodies, and the development of MG. The rationale for this hypothesis is outlined in Table I.

### CONCLUSION

There is considerable circumstantial evidence that the thymus plays a pivotal role in the pathogenesis of MG. Nevertheless, the pathogenic link remains to be forged.

TABLE I Rationale for intrathymic pathogenesis hypothesis

| |
|---|
| nAChRα-reactive CD4+ T cells can be found in the blood of healthy donors as well as MG patients. |
| nAChRα-reactive T and B cells are recovered from MG thymus but not "control" thymus. |
| nAChRα is constitutively expressed on thymic myoid cells and thymic epithelial cells. |
| nAChRα mRNA and MHC class II protein expression on human thymic epithelial cells is upregulated by interferon-γ. |
| Peripheral T cells traffic to thymus where they enter the medulla. |

(Copyright: *Annals of the New York Academy of Sciences*, 998:2003).

We are re-examining the hypothesis that AChR expressed in the thymus drives the pathogenic autoimmune response. We have established a model of intrathymic inflammation that is localized to the thymic medulla and demonstrated that such an inflammatory process promotes the nonspecific entry of peripheral CD4+ T cells into the thymus. We are currently exploiting this model to determine whether (1) AChR-reactive CD4+ T cell homing to the thymus is also augmented by a concurrent intrathymic inflammatory response to an unrelated antigen and (2) AChR-reactive T cell immigrants undergo activation following their engagement of autoantigen in this inflammatory milieu, provide help for the production of anti-AChR antibodies by immigrant autoreactive B cells, and thereby promote the development of a myasthenic syndrome.

### *Acknowledgements*

Studies described in this report were supported by a grant from the Muscular Dystrophy Association and National Institutes of Health grant AI 50058. The authors thank Cecelia Willitt for assistance in preparation of the manuscript.

### *References*

Adelstein, S., Pritchard-Briscoe, H., Anderson, T.A., *et al.* (1991) "Induction of self-tolerance in T cells but not B cells of transgenic mice expressing little self-antigen", *Science* **251**, 1223–1225.

Agus, D., Surh, C.D. and Sprent, J. (1991) "Reentry of T cells to the adult thymus is restricted to activavted cells", *J. Exp. Med.* **173**, 1039–1046.

Alam, S.M., Travers, P.J., Wung, J.L., Nasholds, W., Redpath, S., Jameson, S.C. and Gascoigne, N.R. (1996) "T-cell-receptor affinity and thymocyte positive selection", *Nature* **381**, 616–620.

Anderson, G., Moore, N.C., Owen, J.J. and Jenkinson, E.J. (1996) "Cellular interactions in thymocyte development", *Ann. Rev. Immunol.* **14**, 73–99.

Beeson, D., Morris, A., Vincent, A. and Newsom-Davis, J. (1990) "The human muscle nicotinic acetylcholine receptor α-subunit exists as two isoforms: a novel exon", *EMBO* **9**, 2101–2106.

Berrih-Aknin, S., Arenzana-Seisdedos, F., Cohen, S., Devos, R., Charron, D. and Virelizier, J. (1985) "Interferon-gamma modulates HLA class II antigen expression on cultured human thymic epithelial cells", *J. Immunol.* **35**, 1165–1171.

Blackman, M., Kappler, J. and Marrack, P. (1990) "The role of the TCR in positive and negative selection of developing cells", *Science* **248**, 1335–1341.

Bonomo, A. and Matzinger, P. (1993) "Thymus epithelium induces tissue-specific tolerance", *J. Exp. Med.* **177**, 1153–1164.

Bruno, R., Sabater, L., Tolosa, E., *et al.* (2004) "Different patterns of nicotinic acetylcholine receptor subunit transcription in human thymus", *J. Neuroimaging* **149**, 147–159.

Christadoss, P., Poussin, M. and Deng, C. (2000) "Animal models of myasthenia gravis", *Clin. Immunol.* **94**, 75–87.

Cohen-Kaminsky, S., Delattre, R., Devergne, O., *et al.* (1978) "Synergistic induction of interleukin-6 production and gene expression in human thymic epithelial cells by LPS and cytokines", *Cell Immunol.* **138**, 79–93.

Conti-Fine, B.M., Navaneetham, D., Karachunski, P.L., Raju, R., Diethelm-Okita, B., Okita, D., Howard, J. and Wang, Z. (1998) "T cell recognition of the acetylcholine receptor in myasthenia gravis", *Ann. Acad. Sci.* **841**, 283–308.

Emilie, D., Creven, M.C., Cohen-Kaminsky, S., *et al.* (1991) "*In situ* production of interleukins in hyperplastic thymus from myasthenia gravis patients", *Hum. Pathol.* **22**, 461–468.

Engel, W., Trotter, J.L., MacFarlin, D.E. and McIntosh, C.L. (1977) "Thymic epithelial cells contain acetylcholine receptor", *Lancet* **1**, 1310–1311.

Fuchs, S., Schmidt-Hopfeldd, I. and Tridente, G. (1980) "Thymic lymphocytes bear a surface antigen which cross-reacts with acetylcholine receptor", *Nature* **287**, 162–164.

Fuji, Y. and Lindstrom, J. (1988) "Specificity of the T cell immune response to acetylcholine receptor in experimental autoimmune myasthenia gravis", *J. Immunol.* **140**, 1830–1837.

Galy, A.H. and Spits, H. (1991) "IL-1, IL-4, and IFN-γ differentially regulate cytokine production and cell surface molecule expression in cultured human thymic epithelial cells", *J. Immunol.* **147**, 3823–3830.

Gossmann, J., Lohler, J. and Lehmann-Grube, F. (1991) "Entry of antivirally active T lymphocytes into the thymus of virus-infected mice", *J. Immunol.* **146**, 293–297.

Gowans, J.L. and Knight, E. (1964) "The route of re-circulation of lymphocytes in the rat", *Proc. R. Soc. Lond. B* **159**, 257–282.

Guyon, T., Levasseur, P., Truffault, F., Cottin, C., Ohta, K., Itoh, N. and Ohta, M. (1993) "Nicotinic acetylcholine receptor α subunit variants in human myasthenia gravis: quantification of steady-state levels of messenger RNA in muscle biopsy using the polymerase chain reaction", *J. Clin. Investig.* **94**, 16.

Hirokawa, K., Utsuyama, M. and Sado, T. (1989) "Immunohistological analysis of immigration of thymocyte-precursors into the thymus: evidence for immigration of peripheral T cells into the thymic medulla", *Cell Immunol.* **119**, 160–170.

Hoffman, M.W., Heath, W.R., Ruschmeyer, D. and Miller, J.F. (1995) "Deletion of high-avidity T cells by thymic epithelium", *Proc. Nat. Acad. Sci. USA* **92**, 9851–9855.

Hugo, P., Kappler, J.W., Godfrey, D.I. and Marrack, P.C. (1994) "Thymic epithelial cell lines that mediate positive selection can also induce thymocyte clonal deletion", *J. Immunol.* **152**, 1022–1031.

Iwabuchi, K., Nakayama, K.I., Mc Coy, R.L., *et al.* (1992) "Cellular and peptide requirements for *in vitro* clonal deletion of immature thymocytes", *Proc. Natl Acad. Sci.* **89**, 9000–9004.

Kao, I. and Drachman, D.B. (1977) "Thymic muscle cells bear acetylcholine receptors: possible relation to myasthenia gravis", *Science* **195**, 74–75.

Kisielow, P. and Boehmer, M.V. (1990) "Negative and positive selection of immature thymocytes: timing and the role of the ligand for T cell receptor", *Semin. Immunol.* **2**, 35–44.

Klein, L. and Kyewski, B. (2000) "Promiscuous expression of tissue antigens in the thymus: a key to T-cell tolerance and autoimmunity?", *J. Mol. Med.* **78**, 483–494.

Levinson, A.I. and Wheatley, L. (1995) "The thymus and the pathogenesis of myasthenia gravis", *Clin. Immunol. Immunopathol.* **78**, 1–5.

Levinson, A.I., Zweiman, B. and Lisak, R.P. (1987) "Immunopathogenesis and treatment of myasthenia gravis", *J. Clin. Immunol.* **7**, 187–197.

Levinson, A.I. (2001a) "Myasthenia Gravis", In: Rich, R., *et al.*, eds, Principles and Practice of Clinical Immunology (St. Louis), Vol. 2.

Levinson, A.I., Zheng, Y., Gaulton, S., Moore, J., Pletcher, C.H., Song, D. and Wheatley, L.M. (2001b) "A new model linking intrathymic acetylcholine receptor expression and the pathogenesis of myasthenia gravis", *Ann. Acad. Sci.* **998**, 257–265.

Marshall, D.J. and Gaulton, G.N. (1996) "The role of the immune response in MuLV-induced lymphomagenesis", *Leukemia* **10**, 1860–1866.

Michie, S.A., Kirkpatrick, E.A. and Rouse, R.V. (1988) "Rare peripheral T cells migrate to and persist in normal mouse thymus", *J. Exp. Med.* **168**, 1929–1934.

Mondino, A., Khourts, A. and Jenkins, M.K. (1996) "The anatomy of T-cell activation and tolerance", *Proc. Natl Acad. Sci.* **3**, 2245–2252.

Naparstek, Y., Holoshitz, J., Eissenstein, S., *et al.* (1982) "Effector T lymphocyte line cells migrate to the thymus and persist there", *Nature* **300**, 262–264.

Naparstek, Y., Ben-Nun, A., Holoshitz, J., *et al.* (1993) "T lymphocyte lines producing or vaccinating against autoimmune encephalomyelitis (EAE). Functional activation induces peanut agglutinin receptors and accumulation in the brain and thymus of line cells", *Eur. J. Immunol.* **13**, 418–423.

Naveneetham, D., Penn, A.S., Howard, J.F. and Conti-Fine, B.M. (2001) "Human thymuses express incomplete sets of muscle acetylcholine receptor subunit transcripts that seldom include the δ subunit", *Muscle Nerve* **24**, 203–210.

Oehen, S.U., Ohashi, P.S., Burki, K., Hengartner, H., Zinkernagel, R.M. and Aichele, P. (1994) "Escape of thymocytes and mature T cells from clonal deletion due to limiting tolerogen expression levels", *Cell Immunol.* **158**, 342–352.

Oshima, M., Ashizawa, T., Pollack, M.S. and Atassi, M.Z. (1990) "Autoimunne T cell recognition of human acetylcholine receptor: the sites of T cell recognition in myasthenia gravis on the extracellular part of the α-subunit", *Eur. J. Immunol.* **20**, 2563–2569.

Schluep, M.N., Wilcox, N., Vincent, A., Dhoot, G.K. and Newsom-Davis, J. (1987) "Acetylcholine receptor in human thymic myoid cells *in situ*: an immunologic study", *Ann. Neurol.* **22**, 212–222.

Shevach, E. (2000) "Regulatory T cells in autoimmunity", *Adv. Rev. Immunol.* **18**, 423–449.

Sprent, J., Lo, D., Gao, E.K. and Ron, Y. (1988) "T cell selection in the thymus", *Immunol. Rev.* **101**, 173–190.

Wakkach, A., Guyon, T., Bruand, C., Tzartos, S., Cohen-Kaminsky, S. and Berrih-Aknin, S. (1978) "Expression of acetylcholine receptor genes in human thymic epithelial cells. Implications for myasthenia gravis", *J. Immunol.* **157**, 3752–3760.

Wekerle, H., Ketelson, U.P., Zurn, A.D. and Fulpius, B.W. (1978) "Intrathymic pathogenesis of myasthenia gravis: transient expression of acetylcholine receptors on thymus-derived myogenic cells", *Eur. J. Immunol.* **8**, 579–582.

Westermann, J., Smith, T., Peters, U., *et al.* (1991) "Both activated and nonactivated leukocytes from the periphery continuously enter the thymic medulla of adult rats: phenotypes, sources and magnitude of traffic", *Eur. J. Immunol.* **26**, 1866–1874.

Wheatley, L.M., Urso, D., Tumas, K., Maltzman, J., Loh, E. and Levinson, A.I. (1992) "Molecular characterization of the nicotinic acetylcholine receptor alpha chain in mouse thymus", *J. Immunol.* **148**, 3105–3109.

Wheatley, L.M., Urso, D., Zheng, Y., Loh, E. and Levinson, A.I. (1993) "Molecular analysis of intrathymic nicotinic acetylcholine receptor", *Ann. NY Acad. Sci.* **681**, 74–82.

Zhang, Y., Schluep, M., Frutiger, S., Hughes, G.J., Jeannet, M., Steck, A. and Barkas, T. (1990a) "Immunologic heterogeneity of autoreactive T lymphocytes against the nicotinic acetylcholine receptor in myasthenic patients", *Eur. J. Immunol.* **20**, 2577–2583.

Zhang, Y., Schluep, M., Frutiger, S., Hughes, G.J., Jeannet, M., Steck, A. and Barkas, T. (1990b) "Immunologic heterogeneity of autoreactive T lymphocytes against the nicotinic acetylcholine receptor in myasthenic patients", *Eur. J. Immunol.* **20**, 2577–2583.

Zheng, Y., Wheatley, L.M., Liu, T. and Levinson, A.I. (1999) "Acetylcholine receptor alpha subnit mRNA expression in human thymus: augmented expression in myasthenia gravis and upregulation by interferon-γ", *Clin. Immunol.* **1**, 170–177.

